# Randomized Controlled Clinical Trial of Nanostructured Carbonated Hydroxyapatite for Alveolar Bone Repair

**DOI:** 10.3390/ma12223645

**Published:** 2019-11-06

**Authors:** Rodrigo F. B. Resende, Suelen C. Sartoretto, Marcelo J. Uzeda, Adriana T. N. N. Alves, José A. Calasans-Maia, Alexandre M. Rossi, José Mauro Granjeiro, Mônica D. Calasans-Maia

**Affiliations:** 1 Oral Surgery Department, Dentistry School, Universidade Federal Fluminense, Rua Mario Santos Braga, 28/4º andar, Niterói, Rio de Janeiro CEP 4020-140, Brazil; rodrigoodonto21@hotmail.com (R.F.B.R.); mjuzeda@oi.com.br (M.J.U.); 2 Oral Surgery Department, Dentistry School, Universidade Iguaçu, Avenida Abílio Augusto Távora, 2134, Nova Iguaçu, Rio de Janeiro 26260-045, Brazil; susartoretto@hotmail.com; 3 Department of Oral Diagnosis, Dentistry School, Universidade Federal Fluminense, Rua Mario Santos Braga, 28/4º andar, Niterói, Rio de Janeiro 24020-140, Brazil; 4 Department of Orthodontics, Dentistry School, Universidade Federal Fluminense, Rua Mario Santos Braga, 30/sala 214, Niterói, Rio de Janeiro 24020-140, Brazil; 5 Department of Condsensed Matter, Applied Physics and Nanoscience, Centro Brasileiro de Pesquisas Físicas CBPF, Rua Doutor Xavier Sigaud, 150 Urca, Rio de Janeiro, Rio de Janeiro 22290-180, Brazil; rossi@cbpf.br; 6 Clinical Research Laboratory in Dentistry, Universidade Federal Fluminense, Rua Mario Santos Braga, 28/4º andar, Niterói, Rio de Janeiro 24020-140, Brazil; jmgranjeiro@gmail.com; 7 Directory of Life Sciences Applied Metrology, Instituto Nacional de Metrologia, Qualidade e Tecnologia (INMETRO), Rua Nossa Senhora das Graças, 50-Xerém, Duque de Caxias, Rio de Janeiro 25250-020, Brazil

**Keywords:** clinical trial, xenograft, calcium phosphate, bone loss

## Abstract

The properties of the biodegradation of bone substitutes in the dental socket after extraction is one of the goals of regenerative medicine. This double-blind, randomized, controlled clinical trial aimed to compare the effects of a new bioabsorbable nanostructured carbonated hydroxyapatite (CHA) with a commercially available bovine xenograft (Bio-Oss^®^) and clot (control group) in alveolar preservation. Thirty participants who required tooth extraction and implant placement were enrolled in this study. After 90 days, a sample of the grafted area was obtained for histological and histomorphometric evaluation and an implant was installed at the site. All surgical procedures were successfully carried out without complications and none of the patients were excluded. The samples revealed a statistically significant increase of new bone formation (NFB) in the CHA group compared with Bio-Oss^®^ after 90 days from surgery (*p* < 0.05). However, the clot group presented no differences of NFB compared to CHA and Bio-Oss^®^. The CHA group presented less amount of reminiscent biomaterial compared to Bio-Oss^®^. Both biomaterials were considered osteoconductors, easy to handle, biocompatible, and suitable for alveolar filling. Nanostructured carbonated hydroxyapatite spheres promoted a higher biodegradation rate and is a promising biomaterial for alveolar socket preservation before implant treatment.

## 1. Introduction

Currently, one of the great challenges in dentistry is related to the aesthetic and functional oral rehabilitation of patients, who for congenital, infectious, or traumatic reasons may have an absence of their dental elements associated with alveolar bone loss. After a single tooth extraction, the edentulous site undergoes a bone remodeling with loss of height and width of the alveolar process. [1,2,3,4].

It is known that ridge preservation is important, but what is essential today is the quality of new bone formation at the site of grafting. In a systematic review on the preservation of the alveolar ridge after tooth extraction [5], the authors concluded that alveolar grafting with biomaterial results in a smaller vertical and horizontal reduction of the bone ridges, but found that there is no pattern based on scientific evidence indicating which type of biomaterial should be used.

Alterations in alveolar bone architecture can already be noticed in the first ninety days after the extraction, with a bone loss of up to two-thirds occurring, on average. After one year, a reduction of up to 50% of the vestibular-lingual diameter can be noticed, making it difficult to install a dental implant in this region [4,6,7]. Preservation procedures for the dental socket make the functional restoration of patients possible because the alveolar architecture is maintained due to the impediment of soft and hard tissue collapse in the region, thereby minimizing or even eliminating the need for vertical and/or horizontal increase procedures [8].

Autogenous grafts are an excellent option for bone replacement procedures, with results that have been widely described in the literature, however they have disadvantages that should also be considered, such as complaints of pain at the donor site, increased risk of infection in the graft donor area, insufficient graft for filling large bone losses, and increased surgical time, which contributes to increased patient morbidity and unpredictability regarding the degree of graft resorption [9]. The allogeneic graft is clinically advantageous because it prevents a second surgical procedure, thereby resulting in lower surgical morbidity. However, these materials can generate an exacerbated immune response [10,11]. Another option is the use of xenografts, which are currently widely used for bone repair in the dental socket [6], maxillary sinus floor elevation [12,13], and in periodontal defects [14]. The advantages of xenografts include high predictability, high survival rates, and important preservation of the horizontal and vertical directions of the alveolar walls compared to non-alveolar preservation [15]. However, there are some limitations, such as the low rate of biodegradation due to exposure to thermal treatment, in which the temperature is equal to or higher than the sintering used to remove the organic component from its structure, and the risk of transmitting encephalopathies and other zoonoses, which may lead to increased use restrictions by international health agencies [1].

Among the calcium phosphates (synthetic material) used in dental and orthopedic procedures, hydroxyapatite (HA) stands out due to its physical, chemical, and biological properties. Researchers look to extend the therapeutic capacity of the HA structure by modifying its chemical composition and the size of its crystals, increasing the similarity to natural bone. Although there are excellent biocompatibility and osteoconductive capacity, HA may have limited clinical applications due to its low rate of biodegradation, i.e., it remains for an extended period in the body, occupying space in which neoformed bone tissue should exist [16,17,18].

Carbonate groups constitutes the main ionic substitution in mineral phase of biological apatite [19], demonstrating in previous studies that carbonate-contained hydroxyapatite is more soluble than HA [20,21] and an improvement in the implant/bone interface in [22]. This nanostructured biomaterial exhibits positive changes, such as increased solubility, thermal stability, reduced morphology, and particle size (produced from particles smaller than 100 nm) as well as alterations in the underlying structural characteristics of the HA, improving its biological performance and the capacity of bone binding [21,22,23]. HA particle size and morphology influence the production of inflammatory cytokines—smaller needle-shaped HA particles generated a prolonged inflammatory response compared to spherical shaped nanoparticles—which suggest that these might be detrimental in promoting successful tissue remodeling [24].

Although previous studies have shown the biocompatibility of CHA in pre-clinical [21] and clinical studies [25], the events of bone repair after dental extraction in humans during intervention with CHA microspheres, remain unclear. Based on this background, the purpose of the present study was to compare the effects of bioabsorbable nanostructured carbonated hydroxyapatite (CHA) with a bovine xenograft (Bio-Oss^®^), known to be biocompatible and osteoconductive, and clot (control group) in alveolar preservation.

## 2. Materials and Methods

### 2.1. Synthesis and Physicochemical Characterization of Nanostructured Carbonated Hydroxyapatite

In this study, microspheres (425 to 600 μm) composed of nanostructured carbonated hydroxyapatite containing sodium alginate were prepared using a wet precipitation method containing 6% (by weight) CO_3_, with stoichiometry (1.6 < Ca/P < 2.0). The material was synthesized and characterized in the Laboratory of Biomaterials (LABIOMAT) at the Brazilian Center for Physical Research (CBPF). The CHA was synthesized at 37 °C, without heat treatment (not sintered), thereby maintaining its nanoscale characteristics. The biomaterial was characterized through scanning electron microscopy (SEM—JEOL FEG 250^®^, JEOL, Tokyo, Japan) to examine the morphology of the microspheres and their surfaces. Fourier transform infrared (FTIR) spectroscopy (FTIR—Prestige 21^®^, Shimadzu, Kyoto, Japan) was performed to determine the chemical groups present and the crystalline mineral phases present in the sample, and their crystallinity was examined by XRD (XRD—CuKa Radiation—Zeiss HG4^®^, Carl Zeiss Jena, Germany). The biomaterial was weighed on a precision scale (Unibloc AUY220^®^, Shimadzu, Kyoto, Japan), where each vial was 0.5 g and then packed and sterilized by gamma irradiation (Gammacell 220^®^, Nordion, Ottawa, ON, Canada) 15 kGy/cobalt 60 sample-irradiator at a dose rate of 19.72 Gy/Min for 760 min. The percentage and Ca/P ratio, surface area, pore size and pore volume of CHA and Bio-Oss^®^ (Geistlich Biomaterials, Wolhuser, Switzerland) are presented in Table 1. The specific surface area and porosity of the samples were determined using Micromeritics ASAP 2020 (Micromeritics Instrumental Corp., Norcross, GA, USA) surface area and porosity analyzer equipment at CBPF. Surface area and porosity data were obtained by adsorption of high purity nitrogen gas in the samples. The adsorption process occurred by varying the partial pressure (P/P0) of this gas from 0.05 to 0.998. During the adsorption process, the sample holder containing the sample was immersed in liquid nitrogen at a temperature of about −196 °C. For the evaluation of the calcium and phosphorus molar ratio, a quantitative analysis of the initial calcium was performed by weighing 50 mg of each biomaterial powder and dissolving them in 1 mL of 5% nitric acid in a 50 mL volumetric flask in bidistilled water. A 250 μL aliquot of this solution was transferred to another 50 mL volumetric flask to make a 200× dilution in bidistilled water. Potassium chloride 0.2% was used to suppress ionization at atomic absorption. Released calcium was measured by atomic absorption. The same steps in calcium analysis were followed for the initial phosphorus analysis (Shimadzu, model AA 6800; CBPF Brazil, Rio de Janeiro, Brazil). The calcium–phosphorus ratio was obtained by dividing the calcium value obtained by phosphorus.

### 2.2. Ethical Considerations

This study was performed by the principles outlined in the Helsinki declaration on experimentation involving human beings. All procedures and materials in the present study were previously approved by the Ethics and Research Committee on Human Subjects of the Fluminense Federal University (CEP/HUAP n° 167.103), and the research participants provided written informed consent. Thirty participants (nineteen women and eleven men) were randomly assigned to one of three groups (ten per group) in this present randomized controlled trial, which was performed at the Clinical Research Center of Fluminense Federal University, Niterói, Rio de Janeiro, Brazil (Table 2). The researchers in this study followed a checklist of twenty-two items proposed by the Consolidated Standards of Reporting Trials (CONSORT^®^), aimed at complete and transparent reporting of information to reflect on the quality of the study and enable the practice of ideas advocated by evidence-based practice. The minimum sample size (10 individuals per group) was established in an attempt to minimize publication bias [5,25,26].

### 2.3. Selection of Research Participant

The participants were in good general clinical condition and indicated to undergo unitary dental extraction (for periodontal, trauma or caries problems), without the presence of soft tissue recesses. After diagnosing these teeth for extraction and evaluating these parameters, two different experienced professionals who were not part of this study evaluated the patients according to previously established, specific exclusion and inclusion criteria (Table 3), thus confirming the patients for study participation. The participants were recruited during a six-month period in the oral surgery department at the Dentistry School of Fluminense Federal University, and all subjects were followed for a twelve-month period after their prosthetic rehabilitation. Through a random distribution of envelopes provided by the principal investigator, the blinded participants were assigned to the study groups.

### 2.4. Pre-Surgical Procedures

The medical and dental histories of the participants were reviewed, and each participant was evaluated using periapical radiographs (E-Speed^®^, Kodak, Rochester, New York, NY, USA), clinical photographs (Nikon D3200^®^, Nikon, Tokyo, Japan), study models, and clinical examinations of the extraction sites. Subsequently, the participants were given details about oral hygiene, and a custom surgical splint was made and used during the procedure to obtain greater accuracy at the moment of re-entry for bone biopsies from the center of the grafted area.

### 2.5. Surgical Procedure

The surgical extraction procedure was performed by a single oral and maxillofacial surgeon using a periotome (Quinelato, Rio Claro, São Paulo, Brazil) and suitable forceps (Quinelato, Rio Claro, São Paulo, Brazil), according to the indicated tooth, with the aim of ensuring minor surgical trauma to the surrounding tissues. At the end of the procedure, an alveolar curette (Lucas nº 86, Quinelato, Rio Claro, São Paulo, Brazil) was used for the complete removal of the soft tissue and granulation remains within the wall. Then, a professional who did not participate in this study opened the randomized envelope, and the designated group was revealed only to the surgeon and not to the participant (Group 1: Clot; Group 2: CHA and Group 3: Bio-Oss^®^—Geistlich Biomaterials, Wolhuser, Switzerland). For the participant in whom the biomaterials were installed, the material did not exceed the height of the alveolar crest, and the dental alveolus was completely occupied by the biomaterial, without compaction. Another professional visually inspected the site to determine whether it was saturated with blood. The primary wound closure was performed following mucoperiosteal elevation and rotation of the surgical flap. Azithromycin 500 mg (Astro^®^, Eurofarma, Rio de Janeiro, Brazil) was administered once daily for a period of three days, together with dexamethasone 4 mg (Decadron^®^, Aché, Guarulhos, São Paulo, Brazil), one tablet once daily and Paracetamol 750 mg (Tylenol^®^, Janssen-cilag Farmacêutica, Vila Nova Conceição, São Paulo, Brazil) at a dosage of one tablet every six hours for forty-eight hours. Also, chlorhexidine gel (Perioxidin gel^®^, Gross, Rio de Janeiro, Brazil) was prescribed twice daily for the first two weeks for the application to the surgical area.

Postoperative clinical evaluations of the participants were performed within one, seven, and thirty days to determine the presence of complications such as infection, inflammation, wound dehiscence or loss of graft material. After 90 days, all dental sockets were evaluated through clinical and radiographic examinations.

### 2.6. Surgical Reentry

Ninety days after the extraction, the samples were removed, and the dental implants were installed by a single experienced professional (an implant dentist). Initially, a periapical radiograph was performed to visualize the surgical area. In this procedure, a mucoperiosteal flap was created, and the extraction site was identified using the previously constructed surgical splint. Using a 2 mm diameter surgical trephine drill (SIN, São Paulo, Brazil) at 6 mm deep, the material was obtained from the center of the surgical site using the surgical splint. After this step, the dental implants were installed according to the surgical protocol of the manufacturer. Try-On^®^ or Strong^®^ implants (SIN, São Paulo, Brazil) were used in this study. In the end, the mucoperiosteal flaps were repositioned and sealed using separate stitches with 4.0 silk thread (Ethicon^®^, Johnson & Johnson, Somerville, MA, USA).

### 2.7. Sample Obtention

Bone biopsy samples were obtained from the regions of grafted areas measuring 2 × 6 mm. The collected materials were placed in previously identified flasks containing 4% paraformaldehyde solution buffered at pH 7.4. The samples were kept in the fixative solution for 72 h and then decalcified in EDTA solution (AllkimiaVR^®^, Campinas, São Paulo, Brazil) for 24 h before their embedment. The paraffin blocks were sectioned longitudinally in 5-μm thick slices and then stained with hematoxylin and eosin (HE).

### 2.8. Descriptive Histological Analysis

For the descriptive histological analysis of the slides, a light field light microscope (Olympus BX43^®^, Olympus, Tokyo, Japan) was used. Selected image captures were taken through a microscope-coupled camera (Olympus SC100^®^, Olympus, Tokyo, Japan), coupled with high-resolution software (Cellsens 1.9 Digital Image^®^, Olympus, Tokyo, Japan). A 4× objective was used for a wider view of the area of interest, and 40× was used to obtain greater cellular and tissue details. In each slide, neoformed bone, the biomaterial (when present) and connective tissue were evaluated, and the presence or absence of inflammatory cells, was observed.

### 2.9. Histomorphometric Analysis

Digital images of the HE-stained blades were obtained through a light field light microscope (Olympus, Tokyo, Japan). Six swept fields corresponding to the area of interest were captured with high-resolution software (Cellsens 1.9 Digital Image^®^, Olympus, Tokyo, Japan), increasing the image by 200×. In each photomicrography area corresponding to the new bone formation, reminiscent biomaterial and connective tissue were classified, with the capture interface made available by ImageJ^®^—Version 1.45v software 4.5.0.29 (National Institutes of Health, Maryland, MD, USA). The results were automatically transferred to a Microsoft Excel^®^ (Windows, Albuquerque, NM, USA) spreadsheet for further statistical analysis. The statistical analysis was performed by a single observer, who was unaware of the clinical data.

### 2.10. Statistical Analysis

The new bone formation, reminiscent biomaterial, and connective tissue were performed through the parametric description, with the means and confidence intervals. All data were transformed into a logarithm (y = log y), and the measured variables were evaluated, with a significance level of 5%. The analysis of variance (ANOVA) and Tukey’s post-test was used, with the objective to verify statistical differences in the percentage of new bone formation and connective tissue between groups. The difference in the percentage of biomaterial between the Bio-Oss^®^ and CHA groups was assessed by Student’s *t*-test. A value of *p* < 0.05 was considered to be significant. The analysis was performed using Prism Graph Pad 6.0 software^®^ (Graph Pad Software, La Jolla, CA, USA).

## 3. Results

### 3.1. Analysis of the Tested Biomaterial

**Figure 1:**
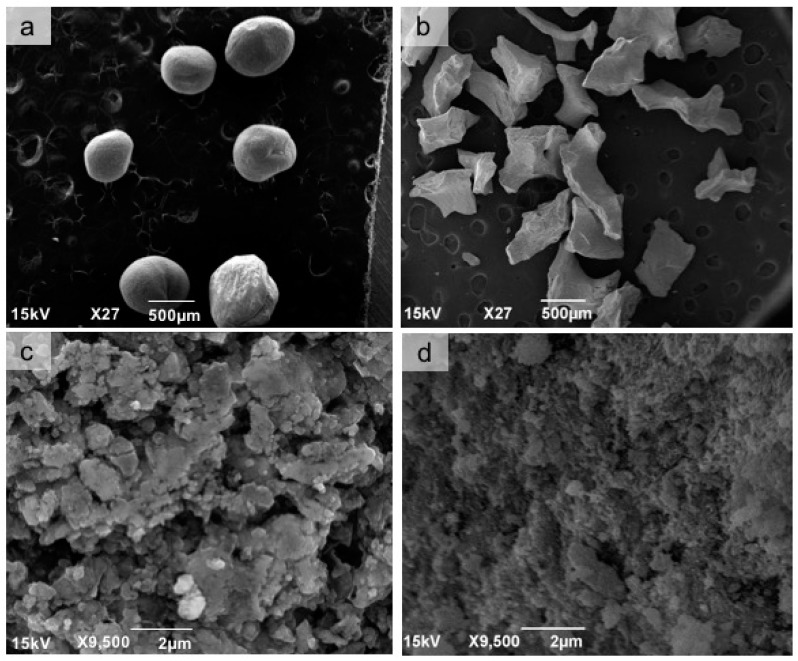
Scanning electron microscopy (SEM) micrographs of the biomaterial groups at 27× magnification, scale bar = 500 µm (**a**,**b**) to observe the morphology of spheres and granules and 9,500× magnification, scale bar = 2 µm (**c**,**d**) to observe the surface parameters: CHA (**a**,**c**) and Bio-Oss^®^ (**b**,**d**).

**Figure 2:**
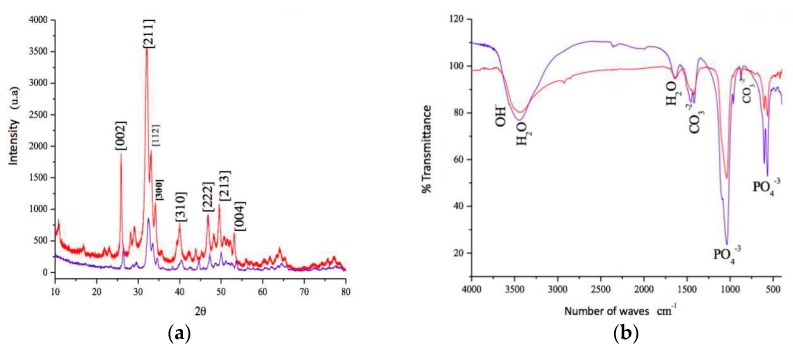
(**a**) X-ray diffraction patterns (XDR) of CHA (red line) and Bio-Oss^®^ (blue line) samples show typical peaks of hydroxyapatite (**b**) Fourier transformed infrared spectra (FTIR) of CHA (red line) and Bio-Oss^®^ (blue line) samples show phosphate bands of hydroxyapatite at 1038, 960, 602 and 560 cm^−1^ and strong carbonate bands in the 1414–1462 cm^−1^ region, due the PO_4_^3−^ for CO_3_^2−^ substitution.

In a scanning electron microscope (SEM), we observed the sphere morphology of the CHA group (Figure 1a) compared to the irregular granules of Bio-Oss^®^ (Figure 1b). At highest magnification, the CHA showed a dense and amorphous surface morphology (Figure 1c), which can be compared with the more regular surface of Bio-Oss^®^ (Figure 1d). In X-ray diffraction, the CHA presented peaks corresponding to standard hydroxyapatite. Elongation can be observed at some peaks, being characteristic of a decrease in the crystallinity of the CHA. This reduction in temperature has a direct influence on the degree of dissolution of the materials (Figure 2a). In the Fourier transform infrared (FTIR) spectroscopy of the sample, bands corresponding to a hydroxyapatite pattern were visualized. Intense and full water bands were observed, as well as carbonates ions, showing that a replacement occurred as expected and also the presence of bands characteristic of phosphate ions (Figure 2b). The percentage of Ca and P, surface area and porous size and volume are presented in Table 1.

### 3.2. Clinical Analysis

Clinically, there were no complications and infections in any of the three groups. No research participants were excluded from the study. Age and sex did not affect the clinical results of this study, and in all groups, complete closure of the surrounding soft tissue was observed within ten days after the tooth extraction. After the 90-day period, when the samples were obtained, the Bio-oss^®^ and CHA groups demonstrated the same bone density during the wrapping observed by the operator. Therefore, the samples were collected, and the implants were installed in all alveolus.

### 3.3. Descriptive Histological Evaluation

**Figure 3:**
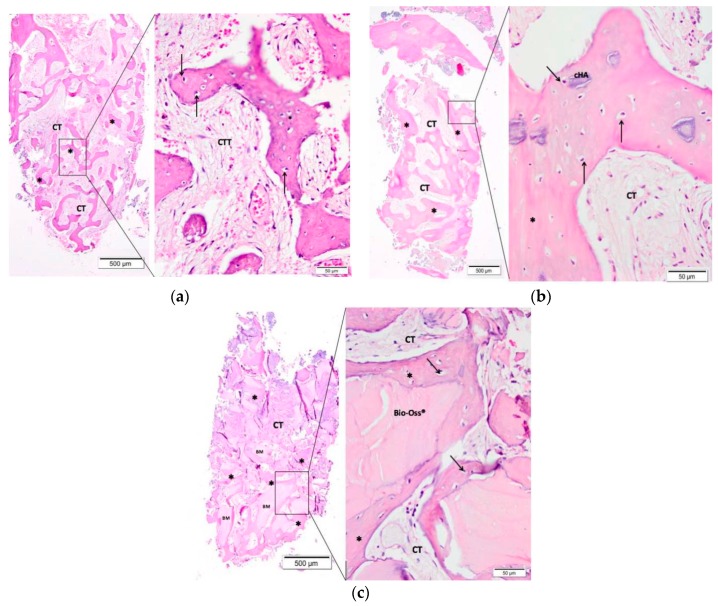
Representative photomicrographs of the alveolar socket after 90 days of biomaterial implantation: (**a**) Control group (Clot), (**b**) CHA group, and (**c**) Bio-Oss^®^ group. The small squares are displayed at 40-fold magnification adjacent to the figures with lower magnification. Connective tissue (CT); (*) new bone formation; osteoblast pavement (black arrow); carbonated hydroxyapatite (CHA) and Bio-Oss^®^. Hematoxylin and eosin-stained.

An experienced pathologist performed a blind histological evaluation. Morphological analysis was performed at light microscopy after hematoxylin-eosin staining. Figure 3 contains the representative photomicrographs of the alveolar socket form each group with 40 and 400x magnification.

Ninety days after the surgical procedure, the clot group showed few trabeculae (the presence of new bone formation) interspersed with connective tissue and few inflammatory cells (Figure 3a).

In the CHA group, a larger amount of new bone formation involving residual fragments of the biomaterial was presented. Also, connective tissue with few inflammatory infiltrates was observed (Figure 3b).

The Bio-Oss^®^ group presented biomaterial of varied sizes and in more quantity than with the CHA involved by new bone formation. Comparatively, the Bio-Oss^®^ and CHA groups presented a similar inflammatory reaction (Figure 3c).

### 3.4. Histomorphometric Evaluation

The means and confidence interval for the parameters of new bone formation, biomaterial, and connective tissue were analyzed three months after implantation.

**Figure 4:**
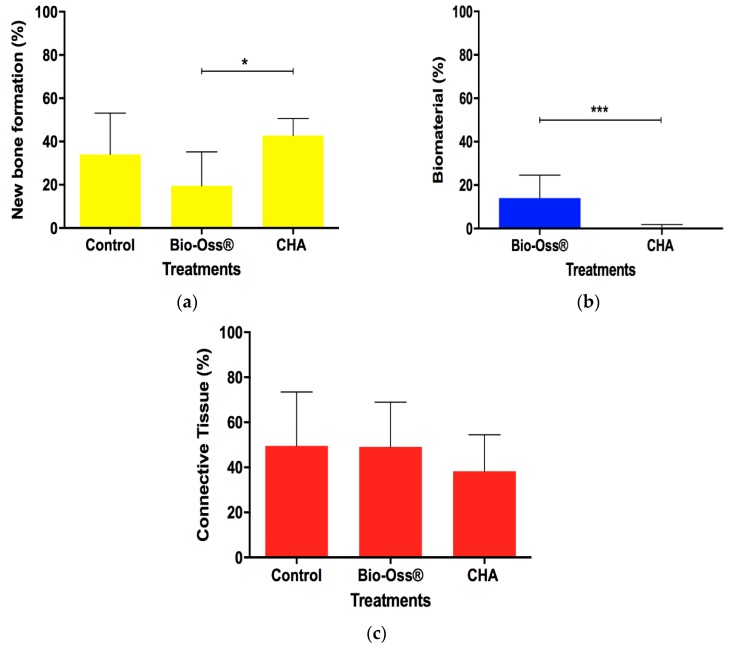
The amount of new bone formation (**a**), reminiscent biomaterial (**b**), and connective tissue (**c**) 90 days after implantation. The horizontal bar represents statistical differences between treatments in the same experimental period. The differences of new bone formation and connective tissue between different treatments were analyzed with two-way ANOVA and Tukey’s post-test (*p* < 0.05) and reminiscent biomaterial with Student’s *t*-test. Results are shown as mean percentages ± confidence interval.

Histomorphometric analysis showed a significant increase of new bone formation (NFB) in the CHA group compared with Bio-Oss ^®^ after 90 days from surgery (*p* < 0.05). However, the clot group presented no statistical difference of NFB compared to the CHA and Bio-Oss^®^ (Figure 4a). The CHA group presented a less amount of reminiscent biomaterial compared to Bio-Oss^®^ (Figure 4b) and there were no differences between groups in the connective tissue parameter (Figure 4c).

## 4. Discussion

The data from this study demonstrated that, clinically, all biomaterials used were adequate for alveolar filling after tooth extraction, in addition to being biocompatible and osteoconductive. This osteoconduction is favored due to the porous environment that is presented by the sphere and granule formats of the used biomaterials [27,28]. The CHA group was developed in spherical form because of the previously confirmed reduction on inflammatory response when compared to the needle shape [24]. The sphere shape shows uniformity, with no borders and angles, which could be considered to be pro-inflammatory agents [24,29,30,31].

Calcium phosphates are one of the most widely used materials for medical and dental applications when bone replacement and regeneration is involved. This popularity is due to the important characteristics demonstrated by this biomaterial, such as biocompatibility with cells and tissues, ability to associate with molecules, ions and metals, greater stability in the biological environment and osteoconductive properties, as well as its versatility in being able to be processed differently [17,32,33]. Among calcium phosphates, HA has been the most studied and used over the past few years due to its physicochemical and biological properties [34]. However, HA and bone substitutes of animal origin are treated at high temperatures, decreasing their rate of dissolution of inorganic compounds and limiting their use; although they are bioactive and osteoconductive, these biomaterials generate a delay in substituting for the formation of newly formed bone in the place where the bone loss occurred.

Modification in the HA structure occurs by the partial substitution of the group, which can occur either in the hydroxyl or phosphate radical, depending on the type of HA. Type A occurs by hydroxyl substitution, and type B through phosphate radical [35]. In this study, a type B carbonated hydroxyapatite was used. According to Landia et al. 2003 [36], the presence of carbonates in the type B HA network generated a decrease in crystallinity and, consequently, an increase in the in vitro and in vivo solubility of the biomaterial, with a higher presence of neoformed bone; these results were also observed by Calasans Maia et al. 2015 [21]. Thus, in the present study, microspheres of non-sintered and non-crystalline carbonated hydroxyapatite were used, which was biodegraded, as observed in the histological analysis 90 days after implantation when compared to the xenograft of thermally treated bovine origin.

Due to this change in its physical–chemical structure, there is greater solubility, thermal stability, and reduced morphology and particle size, as well as alterations in the basic structural characteristics of the HA, thereby modifying the mechanical properties and improving the biological performance and the bonding capacity between the bone and the implant [21,22,37,38]. In previous studies, carbonate has been reported as the main content of the inorganic bone phase, demonstrating that CHA has a greater dissolution than HA [20,32,34].

Another relevant aspect is that CHA is a nanostructured material that is produced from particles smaller than 100 nm, thus, presenting greater similarities with the natural bone form [39,40]. In this study, the material tested consisted of nanometric powders of carbonate hydroxyapatite with crystals of dimensions less than 20 nm. A factor that must be considered, when the biomaterial remains in the alveolus, is the direct relationship between the crystallinity and the temperature of synthesis, to which most of the biomaterials are subjected and which directly reflects the potential of the biodegradation rate. Previous studies have shown that a low temperature of synthesis can improve the surface texture of the material, thereby improving its biodegradability. The higher the crystallinity, the lower the solubility of the biomaterial [21,41]. However, calcination (600, 800, and 1000 °C) or even sintering (1100–1200 °C) increases crystallinity, decreasing its absorption. From the biological point of view, the lower crystallinity could represent a greater facility for fragmentation of the material, resulting in a greater number of particles that are susceptible to phagocytosis [38,42]. This study used microspheres ranging from 425 to 600 μm (not sintered) and synthesized at 37 °C, which, at the end of its experimental time of 90 days, demonstrated its biodegradation, thereby confirming the characteristics of this calcium phosphate compared to those observed in other works using these biomaterials.

In histological and histomorphometric evaluations of this study, the CHA group presented trabeculae of neoformed bone permeating small fragments of biomaterial inside the trabeculae, with intense biodegradation and little inflammatory infiltrate. In the Bio-Oss^®^ group, large fragments of biomaterial that did not undergo biodegradation and that presented a mild inflammatory infiltrate, was visualized. Studies have shown that after a period of 90 or even 180 days, it is still possible to visualize Bio-Oss^®^ xenograft fragments at the surgical site of implantation of this biomaterial because of its sintering, which causes slow degradation [21,43,44].

## 5. Conclusions

After the end of this clinical trial, it was concluded that from the clinical point of view, both experimental biomaterials are easy to handle and suitable for the filling of the dental socket, in addition to demonstrating the properties of biocompatibility and osteoconduction. In all groups, it was possible to install the implants after the experimental period of 90 days of alveolar filling. The CHA and Control group presented a greater amount of new bone formation compared to the Bio-Oss^®^ group and the CHA group presented higher biodegradation compared to the Bio-Oss^®^ group. Although the control group presented bone neoformation comparable to the CHA group and is inexpensive, future studies should be performed to evaluate the thickness and height of the alveolar ridge with and without biomaterial filling.

## Figures and Tables

**Table 1:** Percentage and Ca/P ratio, data on surface area, pore size, and pore volume.

Material	%Ca	%P	Ca/P	Surface Area (m^2^/g)	Porous Size (Å)	Porous Volume (cm^3^/g)
**Bio-Oss^®^**	37, 50	17, 30	1, 68	80, 25	129, 90	0, 26
**CHA**	40, 13	18, 50	1, 53	93, 08	47, 31	0, 11

**Table 2:** List of research participants.

Patient	Sex	Age	Tooth	Experimental Group
1	F	40	25 ^★^	2
2	M	64	14 ^★^	1
3	M	34	36 ^★^	1
4	F	56	27 ^★^	3
5	F	31	24 ^★^	2
6	M	64	36 ^★^	1
7	F	35	26 ^★^	1
8	F	31	25 ^★^	2
9	F	43	15 *	3
10	F	43	25 *	3
11	F	42	11 ^✚^	2
12	F	42	12 ^✚^	2
13	F	56	36 ^✚^	1
14	M	44	12 ^★^	2
15	M	42	26 ^★^	3
16	M	59	16 ^★^	1
17	M	61	15 ^★^	1
18	M	66	25 ^★^	2
19	M	52	36 ^★^	2
20	F	42	36 ^✚^	3
21	M	52	26 ^✚^	2
22	F	56	46 ^✚^	1
23	F	54	13 ^✚^	3
24	M	43	16 ^★^	3
25	F	54	15 ^★^	1
26	F	30	27 ^✚^	1
27	F	54	15 ^✚^	3
28	F	54	23 ^✚^	3
29	F	54	14 ^✚^	2
30	F	54	25 ^✚^	3

* Extraction due to periodontal reason, ^✚^ Caries, ^★^ Tooth/root fracture, 1—Clot, 2—CHA, 3—Bio-Oss^®^.

**Table 3:** Inclusion and exclusion criteria.

Inclusion Criteria	Exclusion Criteria
- Age between 30 and 70 years - Good general health - Presence of a tooth for extraction - An adequate extraction site for the implant installation - A signed consent form	- Use of any medication that may alter or compromise the bone healing response - Smoker - Pregnancy or lactation - Contraindication of surgical treatment - Presenting with a known psychological disorder
